# Efficacy of tranexamic acid in decreasing blood loss during cesarean section

**DOI:** 10.1186/cc12297

**Published:** 2013-03-19

**Authors:** V Zaporozhan, O Tarabrin, D Gavrychenko, G Mazurenko, O Saleh, I Lyoshenko

**Affiliations:** 1 Odessa National Medical University, Odessa, Ukraine

## Introduction

Despite significant progress in obstetric care, the problem of bleeding during labor remains unsolved. Annually in the world, 125,000 women die from obstetric hemorrhage.

## Methods

We performed a randomized, double-blind study in 37 patients who underwent cesarean section. Patients were divided into two groups: the first group (*n = *19) received preoperative (30 minutes before operation) tranexamic acid 10 mg/kg; the second group (*n = *18) received preoperative placebo. The condition of hemostasis was monitored by haemoviscoelastography.

## Results

All patients included in the study before surgery had moderate hypercoagulation and normal fibrinolysis: increasing the intensity of clot formation (ICF) to 11.4% compared with normal rates; the intensity of the retraction and clot lysis (IRCL) was 16.45 ± 1.40 in both groups. At the start of the operation in patients (Group 1), ICF decreased by 9.7% (*P <*0.05), and IRCL decreased by 27.6% (*P <*0.05) compared with preoperatively. In Group 2, there was ICF decrease by 8.8% (*P <*0.05), and IRCL increase by 11.4% (*P <*0.05) compared with preoperatively. At the end of the operation, the condition of hemostasis in both groups came almost to the same value - moderate hypocoagulation, depressed fibrinolysis. In both groups there were no thrombotic complications. Intraoperative blood loss in the first group was 300 ± 40.5 and in the second was 500 ± 60.6.

## Conclusion

Using of tranexamic acid before surgery significantly reduces intraoperative blood loss by 60%, without thrombotic complications.

